# Supplementary material for: Establishing mammalian GLUT kinetics and lipid composition influences in a reconstituted-liposome system
Source: Nat Commun. 2023 Jul 10;14:4070. doi: 10.1038/s41467-023-39711-y (PMC10333360; doi:10.1038/s41467-023-39711-y)
Supplement: Supplementary file 1 — Supplementary Information [file 41467_2023_39711_MOESM1_ESM.pdf]

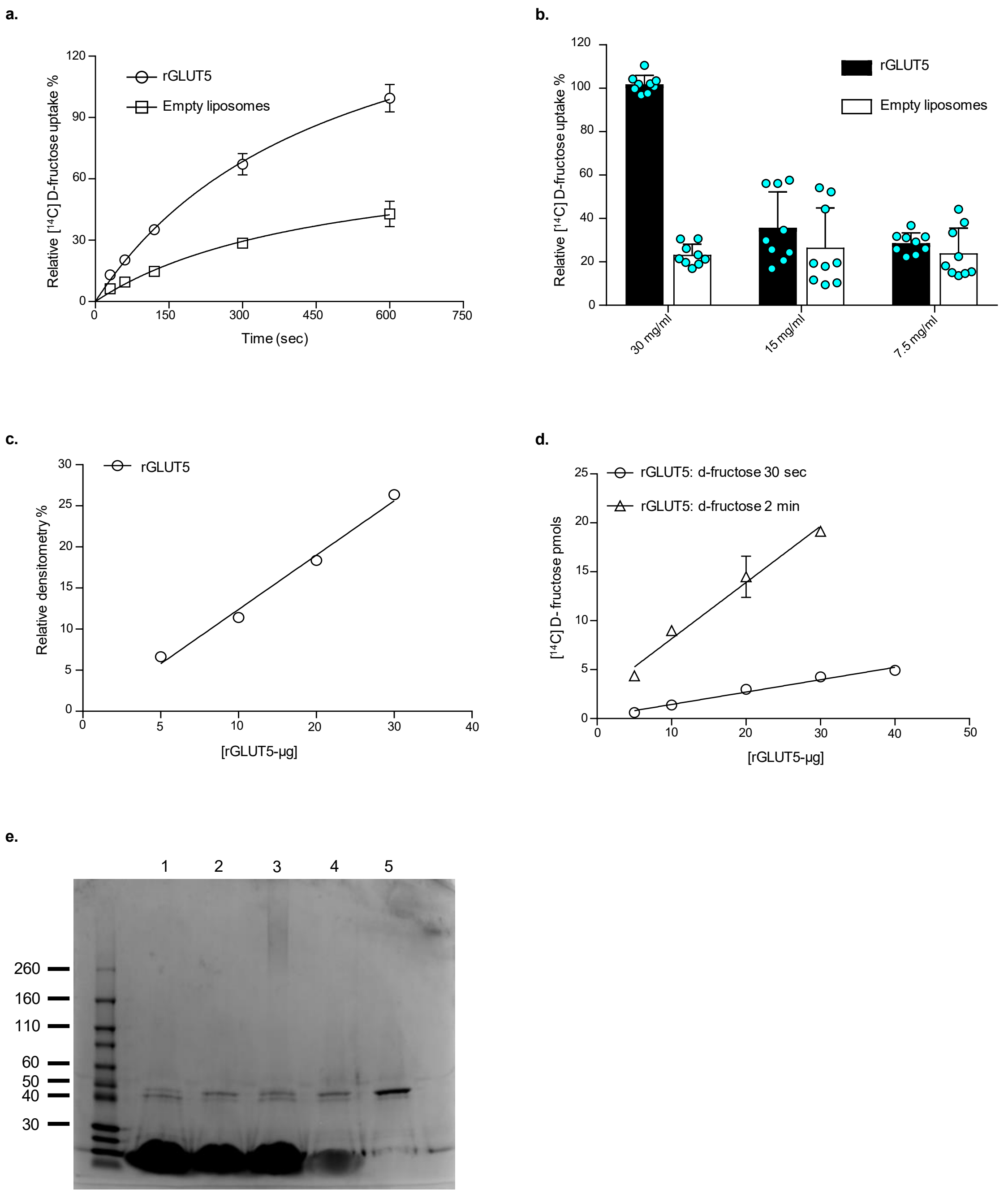

a.
Relative [14C] D-fructose uptake %
rGLUT5
Empty liposomes
Time (sec)
0
150
300
450
600
750
30
60
90
120
b.
Relative [14C] D-fructose uptake %
rGLUT5
Empty liposomes
30 mg/ml
15 mg/ml
7.5 mg/ml
20
40
60
80
100
120
c.
Relative densitometry %
rGLUT5
[rGLUT5-μg]
5
10
15
20
25
30
40
d.
[14C] D- fructose pmols
rGLUT5: d-fructose 30 sec
rGLUT5: d-fructose 2 min
[rGLUT5-μg]
50
e.
1
2
3
4
5
260
160
110
60
50
40
30

Supplementary Fig 1. Optimization of an *in vitro* GLUT transport assay a. rGLUT5 uptake of ^{14}C-D-fructose in liposomes made from bovine-liver lipids. Errors bars indicate the range of 2 independent experiments. **b** rGLUT5 uptake of ^{14}C-D-fructose into proteoliposomes prepared with different concentrations of brain-fraction-seven lipids. Coloring and error bars as in fig 1a. Error bars indicates mean ± s.e.m. of n = 9 independent experiments. **c** Titration of increasing amounts of purified rGLUT5 added for reconstitution into brain-fraction-seven liposomes. On the x axis the amount of rGLUT5 is µg is shown, and y axis indicates relative band densitometry of rGLUT5 (see methods). Non filled circles indicate individual relative densitometry values of reconstituted rGLUT5. All values were normalized against total amount of protein. **d** Titration of increasing amounts of purified rGLUT5 added for reconstitution into brain-fraction-seven liposomes. The x axis represents the amount of rGLUT5 (µg) added to liposomes and y indicates total pmols of ^{14}C-D-fructose transported. Empty circles and triangles represent transport at 30 seconds and 120 seconds, respectively. Error bars indicates mean ± s.e.m. of n = 9 independent experiments. **e** Representative Coomassie-stained SDS-PAGE gel of rGLUT5 in different lipid extracts used to calculate protein reconstitution by densitometry: Lane 1 brain-fraction-seven, 2 Brain I, 3 Soya Lipids, 4 soya PC and lane 5 control of purified rGLUT5 protein. Densitometry was calculated by using the ImageJ software. Purified rGLUT5 (lane 5) was used as reference value corresponding to 100% of theoretical reconstitution. This experiment was done n = 3 of independent experiments.

a.

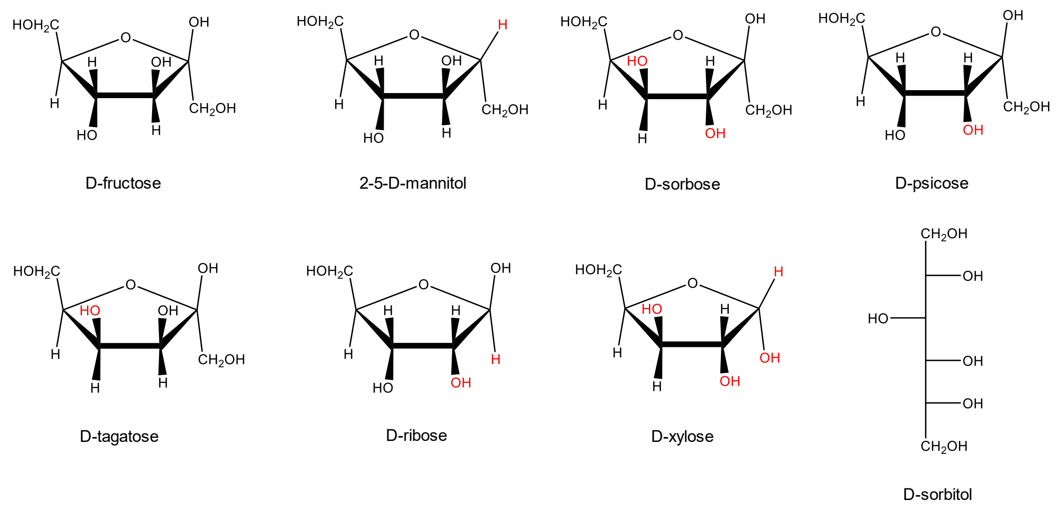
D-fructose
2-5-D-mannitol
D-sorbose
D-psicose
D-tagatose
D-ribose
D-xylose
D-sorbitol

b.

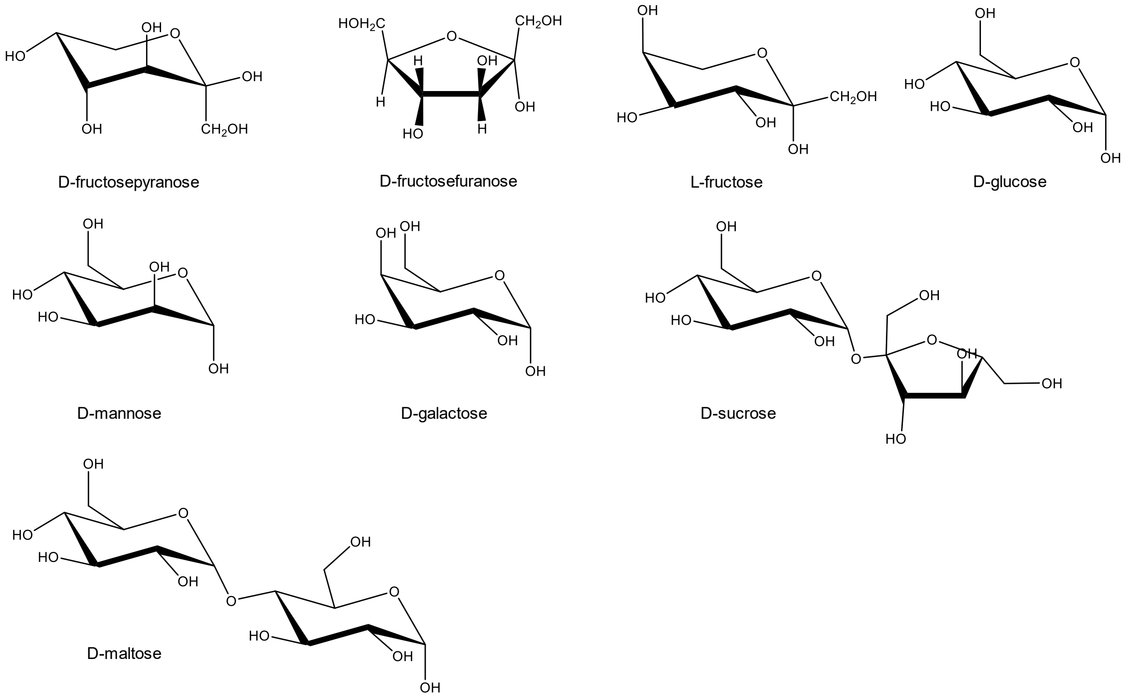
D-fructosepyranose
D-fructosefuranose
L-fructose
D-glucose
D-mannose
D-galactose
D-sucrose
D-maltose

Supplementary Fig 2. Structures of sugars used for competitive uptake of 14C-D-fructose by rGLTU5. **a** Sugars are represented in their pyranose form except for D-fructose, which is in addition shown in the furanose form. **b** Structures of sugars used for competitive uptake in Figure 2b, all sugars are represented in their furanose form for easier comparison, except D-sorbitol. Illustrated in red are all atoms which differ from D-fructose. D-sorbitol is represented in Fischer projection since it does not exist in a cyclic form. All structures were generated using ChemDraw professional (Perkin Elmer).

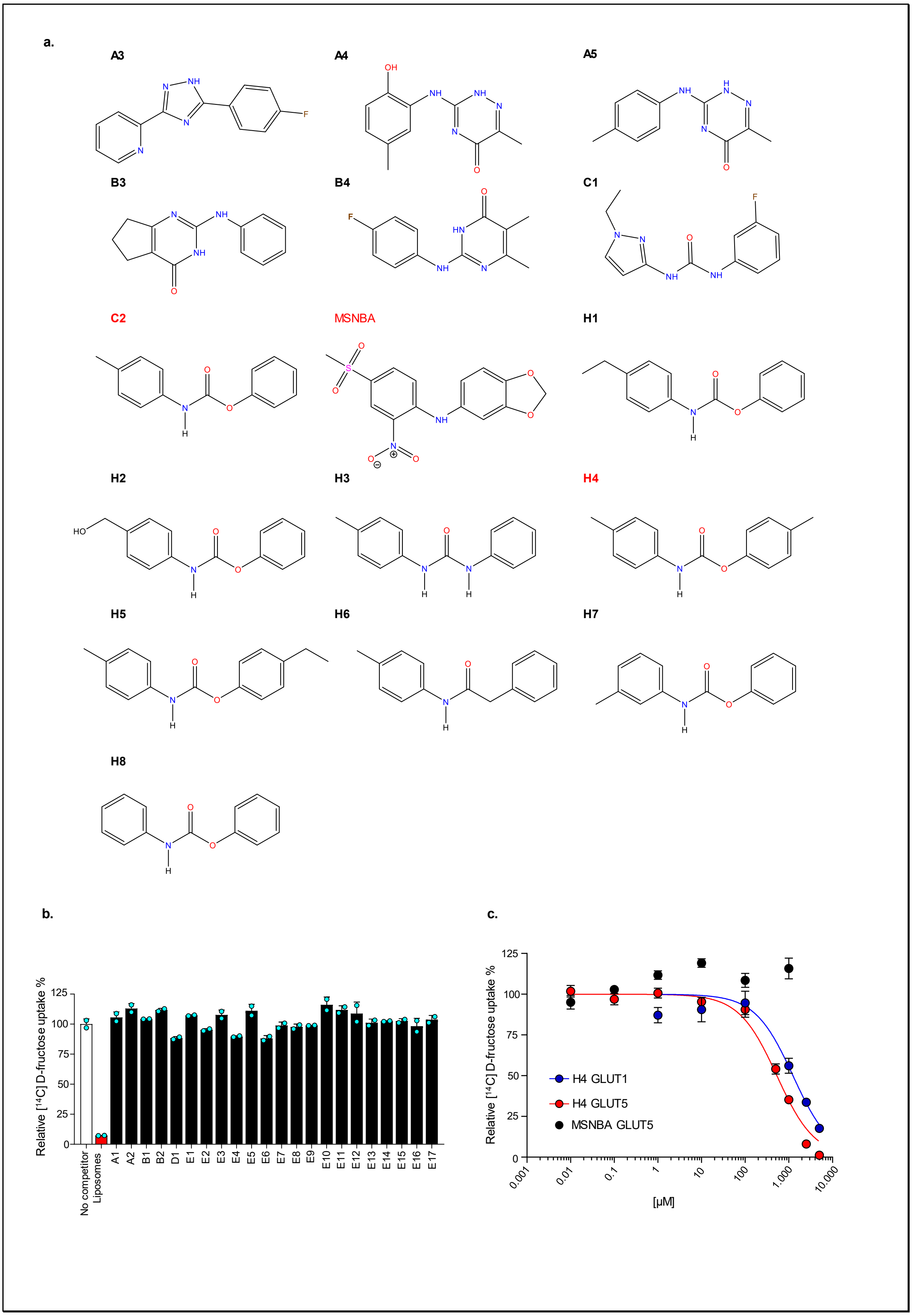

Supplementary Fig 3. Characterization of rGLUT5 inhibitors. a Structure of compounds tested for GLUT5 inhibition in Figure 2d-e. The compounds of particular interest are labelled in red. **b** Competitive uptake of ^{14}C-D-fructose by rGLUT5 in proteoliposomes prepared using brain-fraction-seven in the presence of 100 µM of inhibitors generated from docking that were not shown in Figure 2a.

Following SMILES (Simplified Molecular Input Line Entry System) of each compound represented. A1: Cc1ccc2[nH]c(NCc3ccccc3)nc2c1, A2: COc1ccccc1NC(=O)Nc1ccccc1, B1: COc1ccccc1NC(=O)Nc1cc(C)on1, B2: Cc1nn(C)c2nc(NCc3cc[nH]n3)sc12, D1; O=c1[nH]c(NCc2ccccc2)nc2c1CCC2, E1: O=C(Nc1nnc(-c2ccccn2)[nH]1)C1CCC1, E2: Cc1oncc1NC(=O)Nc1cc(C)ccc1F, E3: COc1ccccc1-c1n[nH]c(NC(C)=O)n1, E4: N#Cc1ccccc1NC(=O)Nc1ccccc1, E5: Cc1ccc(-c2n[nH]c(NC(=O)C3CC3)n2)cc1, E6: Cn1cccc1C(=O)N=c1[nH]c2ccccc2[nH]1, E7: O=C(Nc1n[nH]c(C2CCC2)n1)[C@H]1CC=CCC1, E8: CCC(=O)Nc1nc(-c2ccccc2F)n[nH]1, E9: Cc1ccc(NC(=O)N[C@H]2CCC[C@H]2O)cc1, E10: Cc1c[nH]/c(=N/C(=O)CCc2ccc(C)o2)[nH]1, E11: C[C@H](O)CNC(=O)Nc1ccccc1F, E12: O=C(C[C@@H]1C=CCC1)N=c1[nH]c2ccccc2[nH]1, E13: Cc1n[nH]cc1CNc1nc2ccccc2s1, E14: CN(C)C(=O)CNc1nc2ccccc2s1, E15: C[C@@H](c1ccccc1)N(C)C(=O)c1cn[nH]n1, E16: CN(C)C(=O)CNc1ncnc2c1cnn2C, E17: CCCNC(=O)Nc1cc(Cl)ccc1F. Structure of the respective compounds are shown on Supplementary Fig 4; Error bars indicates the range of two independent experiments. **c** IC_{50} of MSNBA on rGLUT5 (black), 4-methylphenyl- N-(4-methylphenyl) carbamate (H4) on rGLUT5 (red) and hGLUT1 (blue) of proteoliposomes at 5% DMSO. Y axis represents relative [^{14}C]-fructose uptake and x axis concentration of the corresponding inhibitor in μM. Relative D-fructose uptake was normalized after subtracting non-specific transport at the lowest concentration of inhibitor (0.01 μM). Data was fitted using the non-linear function [Inhibitor] *vs* normalized response function in GraphPad prism, fitting was not applicable for MSNBA hence the curve is not shown. H4 IC_{50} of 1.2 ± 0.13 mM and 0.5 ± 0.04 mM were obtained for GLUT1 (blue) and GLUT5 (red) respectively. No inhibition is observed for MSNBA. Error bars indicates mean ± s.e.m. of n = 3 independent experiments.

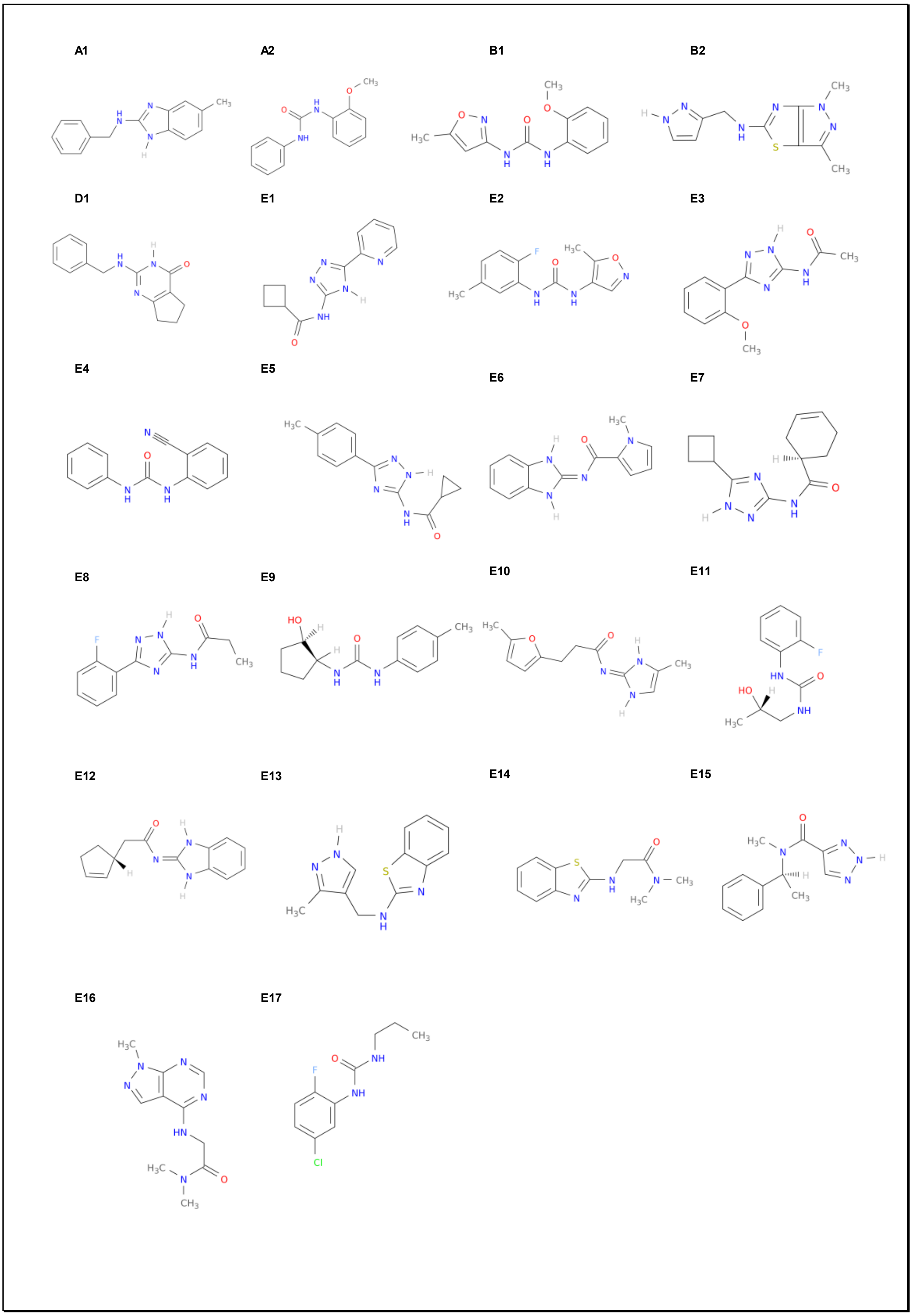

Supplementary Fig. 4. Characterization of rGLUT5 inhibitors. Structures of compounds tested for rGLUT5 inhibition shown in Supplementary Figure 3b.

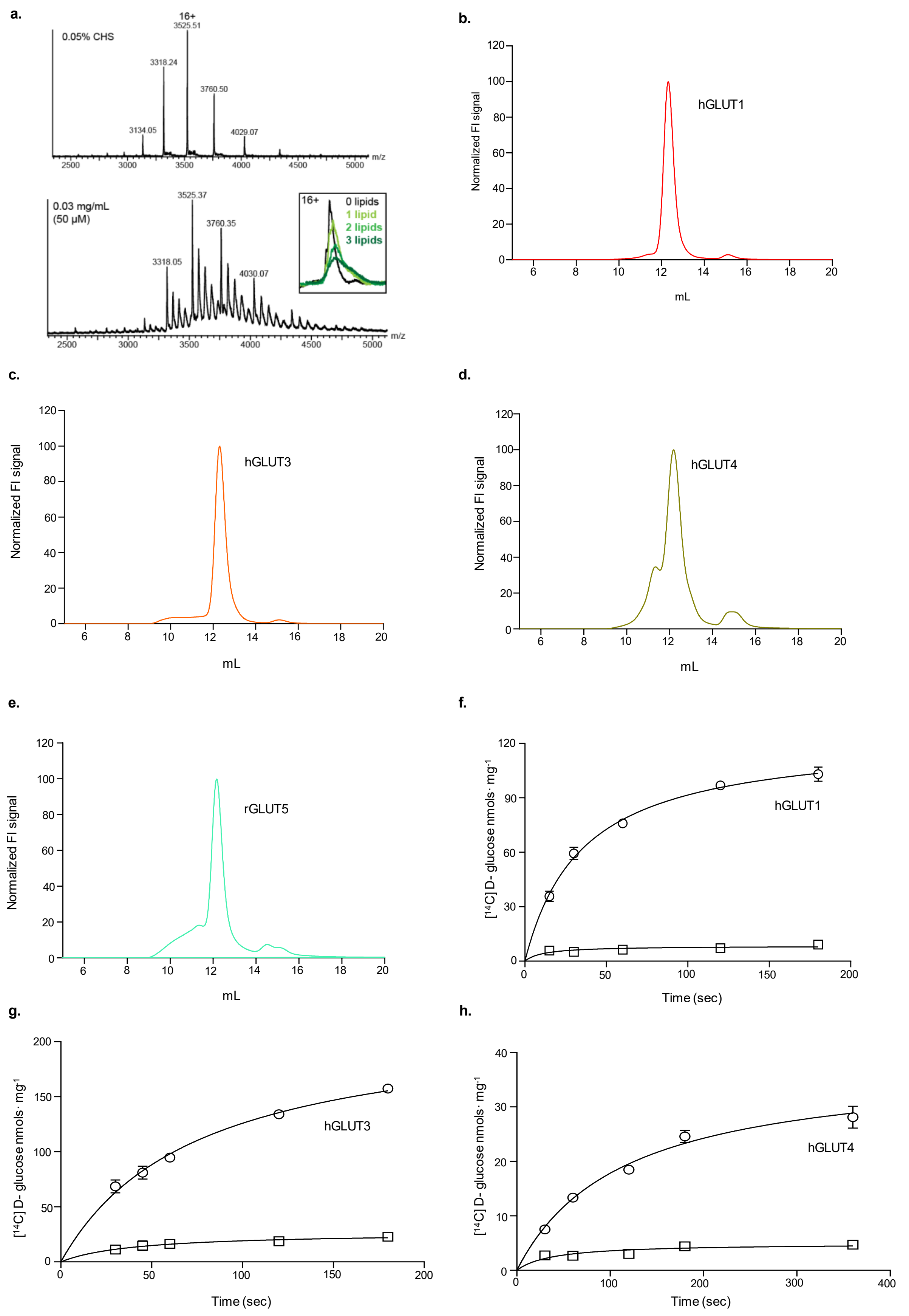

a.
0.05% CHS
16+
3525.51
3318.24
3760.50
3134.05
4029.07
m/z
0.03 mg/mL
(50 μM)
3525.37
3760.35
3318.05
4030.07
16+
0 lipids
1 lipid
2 lipids
3 lipids
b.
Normalized FI signal
hGLUT1
mL
c.
hGLUT3
d.
hGLUT4
e.
rGLUT5
f.
[14C] D- glucose nmols· mg-1
Time (sec)
hGLUT1
g.
hGLUT3
h.
hGLUT4

Supplementary Fig. 5. Native mass spectrometry of rGLUT5 and initial functional characterization of purified human GLUT1, GLUT3 and GLUT4. a Mass spectrometry of purified rGLUT5 in DDM 0.03 % (w/v) in the top panel, with no lipids adducts observed. Addition of lipids from brain-fraction-seven at a concentration of 0.03 mg/mL resulted in the formation of non-specific lipid adducts (lower panel). Overlaying the adduct peaks shows pronounced peak broadening with increasing adduct number, which indicates that that the protein binds a mixture of different lipid species (insert). **b** Fluorescence size exclusion chromatography (FSEC) profile of human GLUT1 pre-equilibrated in their respective buffer (see methods). c As in b., for human GLUT3. **d** As in b., for human GLUT4. **e** As in b., for rGLUT5. **F.** Time course uptake of ^{14}C-D-glucose by hGLUT1 in proteoliposomes and empty liposomes made using brain-fraction-seven represented as empty circles and squares respectively. Errors bars represent s.e.m. of 3 technical repeats. **g** As in f., for human GLUT3. Errors bars represent s.e.m. of 3 technical repeats. **h** As in f., for human GLUT4. Errors bars represent s.e.m. of 3 technical repeats.

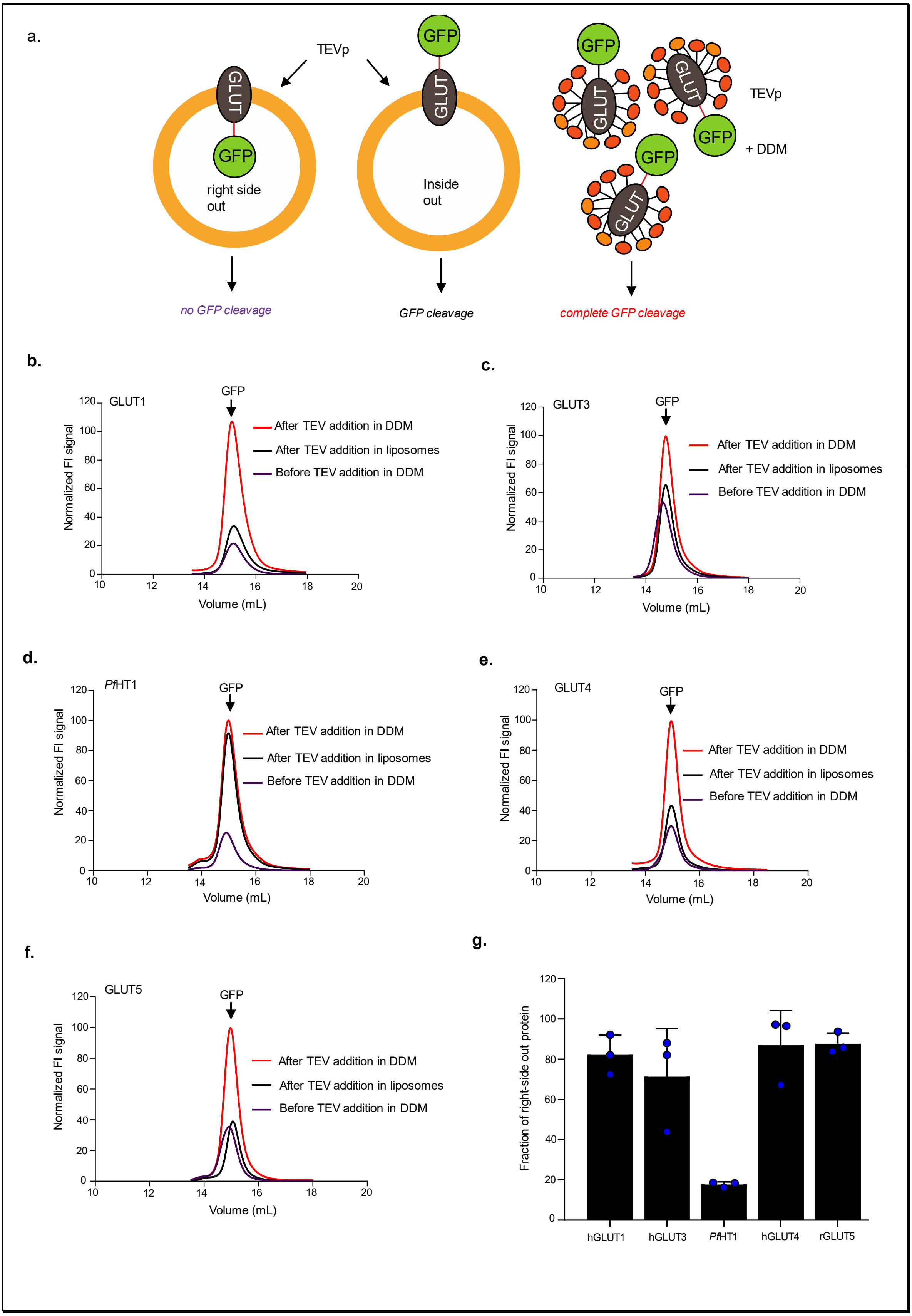
a.
TEVp
GLUT
GFP
right side out
Inside out
TEVp
+ DDM
no GFP cleavage
GFP cleavage
complete GFP cleavage
b.
GLUT1
GFP
After TEV addition in DDM
After TEV addition in liposomes
Before TEV addition in DDM
Normalized Fl signal
Volume (mL)
c.
GLUT3
d.
PfHT1
e.
GLUT4
f.
GLUT5
g.
Fraction of right-side out protein
hGLUT1
hGLUT3
PfHT1
hGLUT4
rGLUT5

Supplementary Fig 6. Calculation of orientation of GLUT and related proteins in proteoliposomes. a Schematic of the approach to estimate orientation in liposomes. Essentially, purified GLUT and *Pf*HT1 proteins were reconstituted as GFP fusions into liposomes and incubated with a TEV protease, which cleaves off the GFP fusion. GLUT and *Pf*HT1 proteins reconstituted with a right-side out orientation are protected from GFP cleavage and the accessible fraction is normalized against total GFP cleavage in the presence of the detergent DDM. **b** Representative Fluorescence size exclusion chromatography (FSEC) traces showing the amount of cleaved GFP for hGLUT1-GFP fusion incubated in DDM buffer w/o TEV (purple trace), hGLUT1-GFP incubation in liposomes with TEV (black trace) and hGLUT1-GFP incubation in liposomes with TEV and DDM (red trace). Note, for sake of comparison, only the peak corresponding to the retention volume of free GFP is shown. Furthermore, under these incubation conditions, we find that even without TEV addition there is cleaved GFP originating from non-TEV proteolysis (purple trace), which has to be accounted for when estimating the additional cleavage with TEV. **c** As in b., for hGLUT3. **d** As in b., for *Pf*HT1. **e** As in b., for hGLUT4. **f** As in b., for rGLUT5. **g** The fraction of right-side our protein in liposomes was calculated based on the amount of additional GFP cleave after TEV addition vs. total cleavage after DDM-solubilization of proteoliposomes. Error bars represent s.e.m. ± mean of n= 3 independent reconstitutions.

	K_M (mM)	V_{max} (µM · min^{-1} · mg^{-1})	k_{cat} (s^{-1})	k_{cat}/K_M (mM · s^{-1})
**GLUT1**	2.0 ± 0.4	4.3 ± 0.3	6.4 ± 0.4	3.1 ± 0.4
**GLUT3**	1.4 ± 0.3	9.0 ± 0.7	12.5 ± 1.0	8.9 ± 1.1
**GLUT4**	8.2 ± 1.1	0.8 ± 0.1	1.1 ± 0.1	0.1 ± 0.0
**GLUT5**	10.9 ± 1.9	52.3 ± 2.9	43.2 ± 2.4	4.0 ± 0.4

Supplementary Table 1. Table of kinetics of hGLUT1-4 and rGLUT5. K_m, V_{max} and k_{cat} were obtained from Michaelis-Menten plot as described before.
